# How and Why Telehealth Interventions Improve Self-Care Among Vulnerable Groups of Patients With Heart Failure: Scoping Review and Rapid Realist Synthesis

**DOI:** 10.2196/78859

**Published:** 2026-02-23

**Authors:** Saleema Allana, Colleen Norris, Armish Hussain, Nada Alaidarous, Raza Muhammad Haider, Alexander M Clark

**Affiliations:** 1 Faculty of Health Sciences, School of Nursing, Western University, 1151 Richmond St, London, ON, Canada, 1 780-935-2820; 2 Faculty of Health Sciences, University of Alberta, Edmonton, AB, Canada; 3 Queen's University, Kingston, ON, Canada; 4 Faculty of Health Disciplines, Athabasca University, Athabasca, AB, Canada

**Keywords:** telehealth, heart failure, marginalized patients, intersectionality, rapid realist review

## Abstract

**Background:**

Heart failure (HF) is a prevalent condition among older adults in Canada, often leading to reduced quality of life and frequent hospitalizations. HF disease management interventions, particularly those delivered through telehealth, aim to improve care by fostering self-care and reducing readmissions. However, disparities in access to and use of HF telehealth services persist among vulnerable populations.

**Objective:**

This study aimed to present the findings of a scoping review and a rapid realist synthesis of HF telehealth interventions for vulnerable groups of patients with HF. This review is underpinned by the metatheory of critical realism and intersectionality theory.

**Methods:**

A rapid realist synthesis of the retrieved literature was undertaken to explore the underlying mechanisms and contexts that make HF telehealth interventions work or not work for marginalized groups of patients with HF.

**Results:**

The realist review findings indicated that vulnerable patients require simple interventions. The findings also suggested that for effective use of telehealth and remote monitoring services, these patients require simplified training that could increase their confidence. The review findings further demonstrated that involving patients’ family members in the delivery of telehealth interventions ensures success.

**Conclusions:**

Future research with vulnerable populations should be underpinned by the critical realism and intersectionality theory and should apply the principles of intersectionality at all stages of the research process, including evaluation and analysis. This review also urges HF practitioners to apply the principles of intersectionality and health equity in clinical practice, such that interventions are simple and personalized, involve family members, include an in-person component, provide training for patients and health professionals, and integrate telemonitoring data.

## Introduction

### Background

Heart failure (HF) is a widespread health condition affecting many older adults in Canada. HF can significantly impair quality of life and is becoming increasingly common as populations age globally [1]. Heart failure disease management interventions (HFDMIs) aim to address these challenges by fostering self-care, enhancing well-being, and reducing recurrent hospitalizations and subsequent health care expenditure. These programs achieve this through patient education, ongoing follow-up, and improving care coordination, which in turn reduces emergency visits and promotes outpatient care [2,3].

HFDMIs encompass a wide range of interventions, including in-person interventions in hospitals, communities, or clinics, as well as remote interventions delivered through telehealth technologies, such as telephone, email, internet, text messages, telemonitoring, mobile health, video consultation, and wireless monitoring systems [4-9]. Despite its potential to improve chronic disease management, telehealth interventions are complex and multifaceted [10]. While telehealth technologies were introduced into HF management in 1998 [5], their complexity as a health delivery model becomes evident when considering the various components and interactions involved and when compared with traditional face-to-face interventions [11]. Effective implementation of telehealth interventions requires harmonizing clinical protocols, leadership support, and technological factors, along with ensuring that patient and health care providers are confident in using technology through education and training [11]. As such, understanding the specific contexts and mechanisms that make telehealth interventions effective in managing HF becomes crucial.

This study details the findings of a rapid realist synthesis, which investigated the use of telehealth solutions for vulnerable patients with HF. The synthesis is framed within the approach of critical realism (CR) [12], aiming to explore the underlying mechanisms and contexts that contribute to the significance of telehealth interventions in improving HF management among vulnerable populations. Additionally, an intersectionality lens was applied to examine how the intersecting social identities of vulnerable groups with HF may affect their access to and use of telehealth services [13].

### Intersectionality, Vulnerability, and Disparities—The Complex Interplay

Intersectionality highlights how different social identities, such as race, gender, age, culture, sexual orientation, socioeconomic status, and geographic location, combine to shape individual experiences [14-16]. These overlapping factors can either grant privilege or deepen inequalities, particularly in health care [14-16]. In the context of HF care, intersectionality plays a vital role in determining access to resources, medical treatment, and self-care support [14]. Patients from marginalized groups, including racialized minority groups and gender minority groups, rural residents, older adults, and people with disabilities, often struggle to receive adequate health care and telehealth services [17]. These disparities stem from systemic barriers such as economic hardship, geographic isolation, and implicit biases within health care systems [17]. The unfortunate reality is that those who require the most care often face the greatest barriers to accessing it, perpetuating health care inequities. These intersecting social identities may act as contextual factors or mechanisms that intensify disparities among marginalized populations and contribute to poorer health outcomes.

### HF Telehealth Interventions—The Inherent Complexity

The use of telehealth has increased during the COVID-19 pandemic [18]. Nonetheless, telehealth adoption varies among demographic groups, with factors such as age, income, race, ethnicity, health literacy, digital skills, and language barriers playing significant roles. These disparities stem from challenges at multiple levels, including the patients themselves, health care systems, telehealth platforms, and overarching policies [18]. Recognizing and addressing these disparities is crucial to ensure fairness in telehealth services, optimize their impact on health care delivery, and ultimately promote telehealth equity [18].

The complexity of HFDMIs lies in the multifaceted nature of the condition, requiring innovative approaches to optimize patient outcomes. The adoption of telehealth interventions for vulnerable patients with HF is complicated by the overlapping social identities that can create barriers to accessing and using telehealth services. Although telehealth became widely accessible during the COVID-19 pandemic [19], these at-risk groups have still faced considerable barriers in making full use of telehealth options [20].

Current understandings of telehealth interventions for HF in vulnerable populations are hindered by 2 issues. First, there is a significant gap in research specifically addressing telehealth solutions tailored to marginalized patients with HF with intersecting identities, limiting insights into their effectiveness [21]. Second, addressing the complexities of telehealth in HF care for disadvantaged groups calls for advanced research methodologies, such as realist methods, that can unravel the contextual elements driving these challenges [22].

### Rapid Realist Synthesis—Exploring Complexity

The use of theory-based research approaches to evaluate telehealth interventions has been urged in the literature [23]. There is increasing consensus that telehealth interventions should be evaluated using complexity-driven approaches [23]. In relation to such interventions, these approaches often bring complexity into various aspects, including the theoretical underpinnings of the research, the type of research questions posed, and the types of methods used. In recent years, the research review and synthesis field has seen an expansion in the variety of approaches used [24]. Among these approaches, realist synthesis has gained importance. Unlike traditional reviews that assess the effectiveness of interventions, realist synthesis aims to comprehend and analyze the underlying mechanisms that contribute to the success or failure of an intervention, offering explanatory insights rather than simple evaluations of its effectiveness [24,25]. Rapid realist syntheses are philosophically and theoretically underpinned by CR.

### The CR Paradigm

CR focuses on uncovering the underlying mechanisms that generate outcomes, making it an explanatory rather than purely descriptive approach. It is particularly suited for addressing “why” questions about social and health phenomena. Within intervention research, this perspective shifts attention from simply assessing effectiveness to examining the reasons behind its effects. In other words, it asks what makes an intervention work in practice. This focus is not only practically valuable but also grounded in CR’s ontological stance [12]. This study used a rapid realist synthesis approach that is underpinned by CR and intersectionality theory, which offers an in-depth understanding of how intersecting vulnerabilities, intervention components, and contextual health care factors interact to shape outcomes in HF telehealth delivery.

## Methods

The search and screening methodology of this review has already been described in a previously published scoping review and intersectionality-based analysis titled “A scoping review and intersectionality-based analysis of heart failure telehealth interventions for vulnerable populations” [26]. The search strategy, screening process, and data extraction were identical to this previous scoping review; full methodological details are previously published in the abovementioned scoping review paper [26]. However, the research questions and the subsequent analysis of data were completely different across the 2 papers. The previous paper reported the findings of a quantitative intersectionality-based analysis of the included studies [26]. However, this paper reports the findings of a rapid realist synthesis, which was undertaken to identify the contexts, mechanisms, and outcomes that make HF telehealth interventions work or not for vulnerable groups of patients with HF. This realist synthesis was important for understanding which HF telehealth interventions work for which vulnerable populations and why.

This scoping review was undertaken following the PRISMA-ScR (Preferred Reporting Items for Systematic Reviews and Meta-Analyses Extension for Scoping Reviews) checklist (Checklist 1). To summarize the search strategy, a systematic search was conducted across MEDLINE, CINAHL, Scopus, and the Cochrane Central Register of Controlled Trials, with additional gray literature sourced via ProQuest Dissertations and Theses Global. Search terms included variations of “heart failure” and “telehealth,” combined with terms representing intersecting identities (eg, race, gender, income, and rurality) to capture studies involving marginalized populations. The screening process used a 2-step, independent, dual-reviewer approach using Covidence (Veritas Health Innovation Ltd), with conflicts resolved by a third reviewer. Studies were eligible if they examined telehealth HFDMIs involving marginalized patients with HF and/or their caregivers. Only full, English-language papers with primary data on the development, implementation, or evaluation of such interventions were included. Outcomes of interest included readmissions, mortality, quality of life, and self-care. Qualitative and mixed methods studies capturing patient, caregiver, or health care professionals’ experiences with telehealth were also considered. Data extraction was guided by a standardized form and included study characteristics, intervention details, and target populations. Study quality was appraised using the Mixed Methods Appraisal Tool.

This rapid realist synthesis was conducted following the Realist and Meta-narrative Evidence Syntheses: Evolving Standards (RAMESES) guidelines for realist reviews, using a theory-driven approach informed by CR and intersectionality theory [27]. Our aim was to uncover how and why HF telehealth interventions work, or do not work, for vulnerable populations by identifying contexts, mechanisms, and their outcomes from published literature.

Unlike stakeholder-engaged realist reviews that involve co-development of program theories through consultation, this rapid realist synthesis is document driven. We did not engage stakeholders in the formulation or refinement of program theories as it was a rapid realist review constrained by time and resources. Instead, the contexts and mechanisms were identified through analysis of peer-reviewed studies that were already mapped in a previously published scoping review [26].

We specifically searched for contexts and mechanisms, identified from the outcomes of the studies, in which HF telehealth interventions worked and did not work. Every study was thoroughly reviewed, analyzed, and summarized to identify these factors. To identify the underlying mechanisms and contexts from the included studies, retroduction was used. Retroduction is a method of analysis in which a pertinent observation or effect is first identified, and then, the researcher moves backward to understand the underlying mechanism or the causal reasoning [28]. Context- and mechanism-related data extracted from the included studies were synthesized by identifying the reoccurring themes across studies.

## Results

### Total Number of Studies Screened and Included

**Figure 1.**
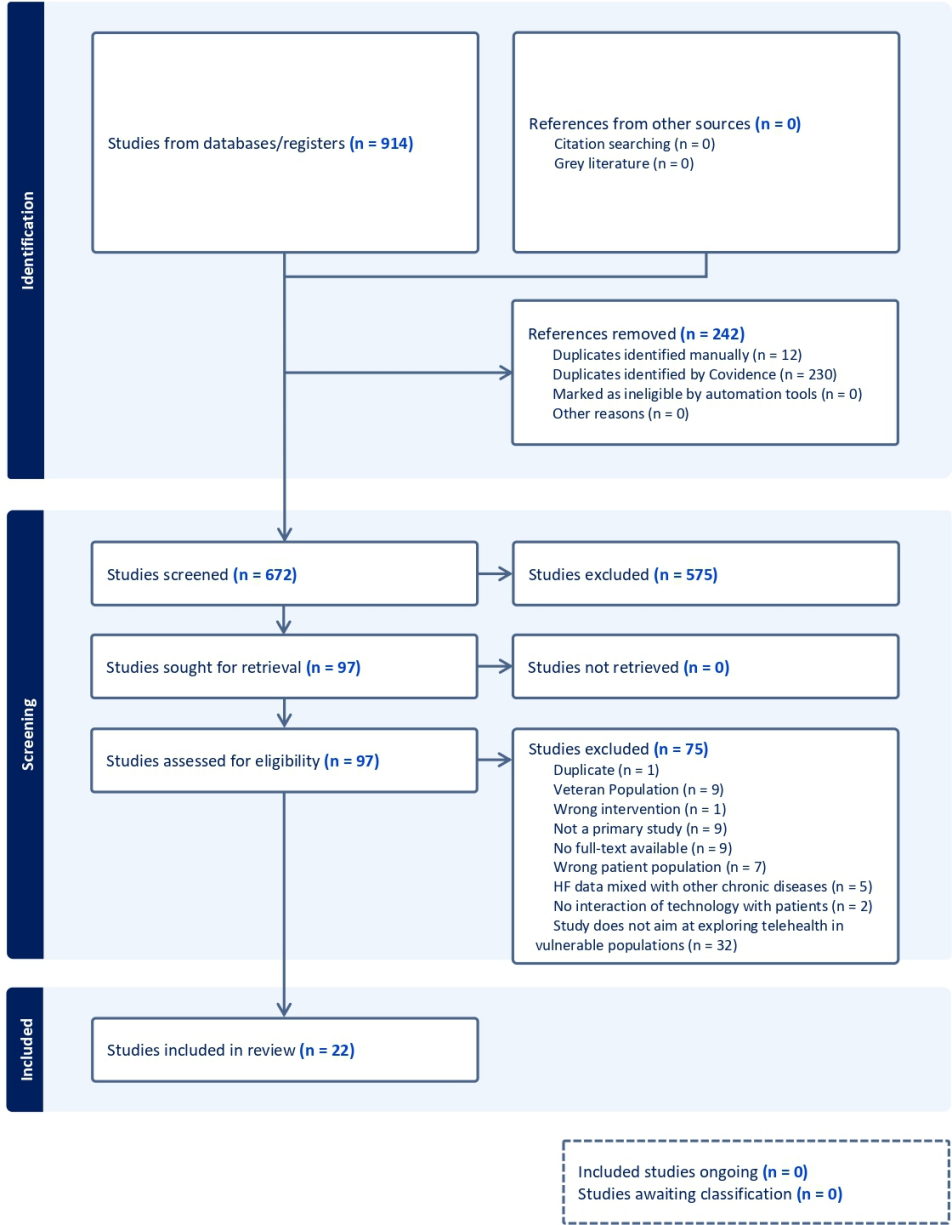
PRISMA (Preferred Reporting Items for Systematic Reviews and Meta-Analyses) diagram. HF: heart failure.

The PRISMA (Preferred Reporting Items for Systematic Reviews and Meta-Analyses) diagram for this scoping review is shown in Figure 1, illustrating the number of studies included, excluded, and screened at each stage of the screening process (Checklist 1).

### Characteristics of the Included Studies

As assessed using the Mixed Methods Appraisal Tool, the studies included in this review were generally of high quality. A diverse range of methodologies and a relatively narrower geographical scope were identified among the 22 studies included in the review. The methodologies varied widely, with 8 (36.4%) studies using randomized controlled trials [29-36]. Other designs included 2 (9.1%) pretest-posttest studies [37,38], 3 (13.6%) cross-sectional or quantitative surveys [39-41], and 3 (13.6%) retrospective data collection and analysis [42-44]. The review also identified 2 (9.1%) feasibility trials [6,45], 2 (9.1%) qualitative studies [46,47], and 1 (4.55%) mixed methods study [48]. Notably, 1 (4.5%) study focused solely on developing and implementing a telehealth program for patients with HF [49]. The studies primarily examined vulnerable groups, including Black [6,29-32,35,42,44,46,47], Hispanic [30,31,33,35,39,42,47], rural [32,34,36,38,40,49], underserved [30,38,41,43], low-income [6,29,37,43-45,47], and low-education patients with HF [6,29,32,44,48]. Telehealth interventions varied, including telephone support [6,29,32,34,36,48], remote monitoring [31,38-40,42,43,45,46,48], video consultations [37,44,49], and combinations of remote monitoring and video consultations [30,47]. Other approaches included telephone case management [33] and a remote monitoring with symptom response system [35]. Among the included studies, 19 (86.4%) were conducted in the United States, indicating that the data predominantly originated from the United States. A total of 10 contexts and mechanisms were identified, of which 7 were contexts and 3 were mechanisms (Table 1).

**Table 1.** Characteristics of the included studies.

Study	Study design	Vulnerable population studied	Type of telehealth intervention
Bakitas et al [6]	Feasibility trial	African Americans, rural and urban population, and low educational level	Telephonic nurse coach sessions and monthly calls
Bakitas et al [29]	Randomized clinical trial	African Americans, rural and urban population, and low educational level	Telephonic nurse coach sessions and monthly calls
Newell et al [49]	Telehealth intervention design and implementation	Rural population	Video consultations
Pekmezaris et al [30]	Randomized controlled trial	Underserved Blacks and Hispanics	Remote monitoring and video consultations
Pekmezaris et al [47]	Qualitative study	Lower-income Blacks and Hispanics	Remote monitoring and video consultations
Dang et al [31]	Randomized controlled trial	Blacks and Hispanics	Mobile phone–based telemonitoring
Dionne-Odom et al [32]	Randomized clinical trial	African Americans, rural and urban population, and low educational level	Telephonic nurse coach sessions and monthly calls
Rosen et al [37]	Pretest and posttest design	Low-income patients	Video consultations
Riegel et al [33]	Randomized controlled trial	Hispanics of Mexican origin	Telephone case management, including decision support software and telephone support
Turchioe et al [39]	Cross-sectional feasibility study	English- and Spanish-speaking older adults	Mobile app to report symptoms
Heiney et al [46]	Qualitative study	African Americans	Mobile app for weight monitoring, health messages, and journaling
Jaana and Sherrard [40]	Cross-sectional study	Rural population	Remote monitoring via mobile phone
Bakhshi et al [45]	Feasibility trial	Low-income patients	Remote monitoring
Liu [41]	Quantitative survey method	Medically underserved urban population	Telemonitoring
Srisuk et al [34]	Randomized controlled trial	Rural population	Telephone support
Cabán [42]	Retrospective quantitative study	Hispanics, non-Hispanic Blacks, and non-Hispanic Whites	Home telemonitoring
Soran et al [35]	Randomized controlled trial	Older women, African Americans, and Hispanics	Telemonitoring and telephone-based symptom response system
Riley et al [38]	Pretest-posttest design	Underserved patients in rural communities	Remote monitoring
Krum et al [36]	Randomized controlled trial	Rural and remote communities of Australia	Telephone support
Lefler et al [48]	Mixed methods study	Low educational level	Remote monitoring and telephone support
Davis et al [43]	Retrospective cohort design	Underserved patients	Remote monitoring
Sammour et al [44]	Retrospective study design	African Americans, low-income, and low educational level	Video and telephone consultations

### Contexts That Made HF Telehealth Interventions Work

#### Family Members’ Involvement in Telehealth or Remote Monitoring Programs

The involvement of family members in training and implementing telehealth or remote monitoring programs was found to be crucial for the success of interventions. Studies by Riley et al [38] and Pekmazaris et al [47] demonstrated the positive impact of including family members in training and support. A study conducted in rural Thailand exclusively tested a family-based telehealth intervention and found it to be highly successful [34]. Similarly, Bakitas et al [6,47] and Dionne-Odom et al [32] tested palliative care interventions for patients with HF that involved the active participation of family caregivers and demonstrated success.

#### Training of Health Professionals

The importance of training patients, family caregivers, and health professionals for telehealth programs has been emphasized in the review findings [6,31,35,37]. Bakitas et al [6] provided 20 hours of training to nurse coaches, focusing on effective communication and patient management through telehealth intervention. Dang et al [31] strongly recommended staff training based on their experience with telehealth interventions involving Black and Hispanic patients with HF. A different study observed that primary care physicians did not respond appropriately to clinical deterioration in remotely monitored patients with HF [35]. Accordingly, Soran et al [35] recommended training physicians and other health professionals involved in telehealth and remote monitoring interventions to ensure prompt and appropriate actions. In an interesting telehealth intervention by Rosen et al [37], social workers played a primary role in delivering the intervention. They received extensive training in health-related aspects of HF care while contributing their expertise in social aspects of care.

#### Significance of an In-Person Component Along With the Telehealth or Remote Monitoring Program

One of the significant findings of this review was that, in many studies, participants appreciated or requested at least 1 in-person meeting or interaction with their health care providers along with a telehealth or remote monitoring component. Those interventions that were a combination of both in-person and telehealth components were also found to be relatively more effective [6,32,34,37,43,47]. The in-person components, in most cases, were meant to build rapport with patients and support training or education. In the study by Pekmazaris et al [ 47], the Community Advisory Board, consisting of low-income Black and Hispanic patients, recommended arranging the first in-person meeting between patients with HF and the telemonitoring nurse. Comparing their study findings with other studies in this area of research, Soran et al [35] strongly suggested including a home visit, even if it was only possible once.

#### One Size Does Not Fit All—Personalized Tailoring of Telehealth Interventions

In this review, the importance of personalized and tailored interventions for vulnerable groups of patients with HF has been highlighted through several studies [31,33-35,39,43,46]. Riegel et al [33] conducted a trial of telephone case management with Hispanic patients with HF of Mexican origin, customizing the intervention to align with their cultural needs. Bilingual and bicultural health professionals, along with the integration of cultural values, trust, family inclusion, and problem-solving, were key components of this tailored intervention. Another example was the HF telehealth intervention by Srisuk et al [34] for rural Thai patients, which involved family caregivers at each stage of the intervention and incorporated cultural elements such as translated materials and culturally relevant content. Pekmazaris et al [47] used a community-based participatory research approach, involving Black and Hispanic patients with HF in developing a user-centered intervention through a Community Advisory Board. Patient feedback was used to modify the intervention delivery and equipment. Davis et al [43] recommended personalized, consistent feedback for patients with HF by telehealth providers (physicians, nurses, and allied health professionals involved in the delivery of telehealth services) based on their findings, while Dang et al [31] allowed patients to choose their preferred time and language for daily questions, emphasizing the importance of personalization. Heiney et al [46] involved African American patients in developing a mobile health intervention and ensured that the content was culturally relevant to their values. Pekmazaris et al [47] also emphasized the unique characteristics of vulnerable patients with HF, such as younger age and higher comorbidity rates, necessitating tailored telehealth programs considering cultural values, beliefs, and language.

#### Contextual Realities of Health Care Systems

Telehealth interventions are performed within the broader health care system; therefore, these are bound to be affected by the contextual realities of health care systems. In a study by Lefler et al [48], participants alluded to the unavailability of health professionals to respond to them or to unsatisfactory responses by them, when patients needed guidance on managing their symptoms. This could potentially lead to clinical deterioration and could result in the very outcomes that we are trying to prevent through the use of telehealth interventions. Dang et al [31] suggested using more health professionals and a variety of them (nurses, doctors, and dieticians) to be better able to support patients with HF via telehealth interventions.

Another very important contextual reality has been discussed by Soran et al [35]. They indicated the fact that a typical patient with HF is not seen by a cardiologist or a chronic care team; rather, such patients often present to primary care physicians in community settings. This holds true for most vulnerable patients with HF. However, most disease management programs, including telehealth interventions, are run in large academic centers [35]. To reach vulnerable patient populations, HF telehealth interventions need to be tailored accordingly and must be offered in community settings via primary care physicians’ offices.

Soran et al [35] also alluded to another important fact that is part of the context. They indicated that when telehealth interventions are not integrated into workflow practices, it becomes difficult to run these interventions successfully. Riley et al [38] also made important recommendations in this regard. They suggested that to be useful, telemonitoring data must be incorporated in the care team’s workflow, such that health care providers (physicians, nurses, and allied health professionals) know when and how to intervene based on the data received from patients via telemonitoring [38].

#### Equipment-Related Issues

As part of this review, some studies have highlighted the equipment-related issues that interfered with the delivery of telehealth interventions. Most often, these issues were highlighted by the patients with HF themselves. Some participants described setting up the equipment as a tough task, such as leveling the weighing scale, setting up the iPad, etc [48]. In another study by Dang et al [31], Black and Hispanic patients with HF suggested having larger font sizes on screen, accurate translation of equipment names in Spanish, slowing down the speed of verbal instructions provided during telemonitoring, and decreasing the use of medical jargon.

#### Technical Issues

As telehealth and remote monitoring interventions largely rely on internet connectivity for virtual consultations and data transmission, internet connectivity problems emerged as one of the biggest technical issues that hampered the delivery of telehealth and remote monitoring interventions [38,47]. Riley et al [38] reported working proactively to ensure adequate network stability required for the transmission of patient data. Another important technical issue was discussed by Dang et al [31] based on their experience with web browser messaging. They suggested that text messages are not only more user-friendly than web browser messages but also require less data and therefore pose fewer connectivity challenges.

### Mechanisms That Made HF Telehealth Interventions Work

#### Simplified Interventions Work vs Too Complex

Simplified interventions have been found to be more effective for HF telehealth interventions targeting vulnerable patient populations, considering their low levels of education, health literacy, and digital literacy [38,45,48]. Riley et al [38] emphasized the complexity of remote monitoring interventions for rural patients, while Lefler et al [48] highlighted the cognitive changes and poor health literacy among patients with HF. Bakhshi et al [45] advocated for simplicity in telehealth interventions, ensuring the automatic transmission of weight data for ease of remote monitoring.

#### Simplified Patient Training Increases Confidence for the Use of Telehealth and Remote Monitoring Equipment

Simplified patient training plays a crucial role in increasing confidence and proficiency in using telehealth and remote monitoring equipment [6,41,42,47]. Cabán [42] provided basic training to racialized groups, teaching them essential skills and electronic communication with health care providers (physicians, nurses, and allied health professionals). Dang et al [31] offered one-on-one instruction and visual manuals for participants with low educational attainment, while another study emphasized the need for more visual training materials [6]. Additionally, lower-income and racialized minority groups and gender minority groups with HF suggested increasing training time and offering in-person sessions to enhance the effectiveness of the telehealth program for these patients [47].

#### Sense of Security and Increased Comfort With Managing Symptoms Reinforces the Use of Telemonitoring

An increased sense of security and comfort in managing symptoms was reported as positive outcomes of telemonitoring interventions, reinforcing their use [41,43,48]. For instance, in a study by Lefler et al [48], a theme emerged, “watching over me,” whereby participants described a sense of confidence that emerged with telemonitoring. This confidence came from the feeling that someone experienced and knowledgeable in health care was watching over them, leading them to use telemonitoring more effectively. Participants in another study discussed a similar sense of security [43]. Another important theme in the Lefler et al [48] study was the increased comfort in managing HF symptoms and heightened awareness of one’s health experienced by patients who used telemonitoring regularly. This comfort and awareness reinforced their use of telemonitoring daily. Liu [41] suggested that patients with HF who are confident and proficient in using technology and who perceive it as easy to use are more likely to view telehealth interventions as useful.

## Discussion

### Principal Findings

As per the findings of the previous paper, there was a lack of integration of the intersectionality-based principles in the body of literature on HF telehealth use among vulnerable populations [26]. To build upon these findings, we wanted to explore which of these interventions worked or did not work for these vulnerable populations and how and why they worked or did not work. Therefore, a realist synthesis approach was used to build on the findings of the initial review. This rapid realist synthesis outlined important contextual factors, including family involvement, health professional training, in-person components, personalized tailoring, contextual realities and systems factors, equipment, and technical issues. The synthesis also highlighted mechanisms that can improve HF telehealth programs for vulnerable patient populations. It recognized the effectiveness of simplified interventions, including patient training, which can lead to positive outcomes such as (1) increased patient confidence and proficiency in using telehealth technologies and (2) enhanced sense of security and comfort in managing symptoms and overall health.

To further strengthen the analysis, we considered the application of intersectionality in designing, implementing, and evaluating HF telehealth programs for vulnerable populations. The review highlights the need for increased research attention to health inequities experienced by these populations, particularly in designing interventions across different countries and vulnerable or marginalized groups. This aligns with other research highlighting the importance of using various research methods, including qualitative, interpretive, or realist approaches, to comprehensively understand complexities and mechanisms of these interventions [12].

The analysis in this review indicates that while studies have focused on specific populations such as African Americans, Hispanics, and rural low-income groups, there is a need to include other vulnerable populations, including other racialized minority groups and gender minority groups, immigrants and refugees, women with intersecting social identities, and Indigenous peoples. Additionally, the review highlights the effectiveness of various telehealth interventions such as telephone support, video consultations, remote monitoring, and case management, emphasizing the importance of tailoring these interventions to meet the specific needs of vulnerable patients with HF.

Access and use inequities in telehealth services for vulnerable patients are significant, given their low educational, health literacy, and digital literacy levels [50]. Simplified interventions and training are essential to enhance their confidence and proficiency in using telehealth and remote monitoring equipment [50]. Family involvement, particularly in racialized minority groups and rural populations, is crucial for success, considering the cultural significance of family in patient care [33,34].

The review also highlights the importance of health professionals’ training in delivering effective telehealth interventions [51]. Ensuring access to HF telehealth programs for vulnerable patients, particularly within primary care clinics in community settings, is essential to maximize their benefits [12].

In-person interactions and the personalization of interventions are valued by patients, emphasizing the significance of building rapport and tailoring interventions to cultural, linguistic, and contextual needs [52]. Integrating telemonitoring data into the care team’s workflow enables prompt action and decision-making in case of clinical deterioration [12].

Communication-related issues, such as language translations, avoiding medical jargon, and using simple and understandable language, are crucial for effectively using telehealth interventions by racialized minority groups and individuals with lower health literacy [12]. The review also highlights the positive impact of telemonitoring interventions on patients’ confidence, sense of security, and comfort in managing HF symptoms, reinforcing their use [12].

### Strengths and Limitations

This review is notable for its originality, being the first to conduct a rapid realist synthesis in this research area, and its systematic search in multiple and multidisciplinary databases. However, it is limited by its reliance on published articles in English, the exclusion of gray literature, and the inability to collect primary data from key stakeholders, which could have enhanced the findings and theory generation.

### Recommendations

The review findings offer valuable insights for improving HF telehealth interventions in clinical practice and research. Key practice-based recommendations include simplifying telehealth interventions for patients with lower education, health literacy, and digital literacy; providing tailored training; involving family members for support; training health care professionals; incorporating in-person components; personalizing interventions; offering telehealth in community settings; integrating telemonitoring data; building trust and acceptance for self-management; and using clear language and appropriate translations for racialized minority groups.

### Conclusions

In conclusion, this scoping review highlights the need for greater incorporation of appropriate philosophical and theoretical foundations in research on HF telehealth interventions for vulnerable populations. While intersectionality principles have been applied in problem identification and intervention development, their application in evaluation stages needs to be improved. In addressing this, future research should adopt CR and intersectionality theory and apply intersectionality principles throughout the research process. The review emphasizes the importance of HF practitioners applying intersectionality and health equity principles in clinical practice by implementing simple, personalized interventions involving family members; including an in-person component; training patients and health professionals; and integrating telemonitoring data into care workflows. By fully implementing these findings, HF telehealth interventions can significantly improve care for vulnerable patients, addressing their specific needs; enhancing access, acceptance, and use of telehealth; and ultimately reducing health inequities.

## Supplementary material

10.2196/78859 Checklist 1 PRISMA-ScR checklist.

## Abbreviations

CR: critical realism
HF: heart failure
HFDMI: heart failure disease management intervention
PRISMA: Preferred Reporting Items for Systematic Reviews and Meta-Analyses
PRISMA-ScR: Preferred Reporting Items for Systematic Reviews and Meta-Analyses Extension for Scoping Reviews
RAMESES: Realist and Meta-narrative Evidence Syntheses: Evolving Standards
